# The live cell DNA stain SiR-Hoechst induces DNA damage responses and impairs cell cycle progression

**DOI:** 10.1038/s41598-018-26307-6

**Published:** 2018-05-21

**Authors:** Onur Sen, Adrian T. Saurin, Jonathan M. G. Higgins

**Affiliations:** 1 Cell Division Biology Group, Institute for Cell and Molecular Biosciences, Newcastle University, Medical School, Framlington Place, Newcastle upon Tyne, NE2 4HH UK; 2 Division of Cancer Research, School of Medicine, Jacqui Wood Cancer Centre, Ninewells Hospital and Medical School, University of Dundee, Dundee, DD1 9SY UK

## Abstract

SiR-Hoechst (SiR-DNA) is a far-red fluorescent DNA probe being used widely for time-lapse imaging of living cells that is reported to be minimally toxic at concentrations as high as 10–25 µM. However, measuring nuclear import of Cyclin B1, inhibition of mitotic entry, and the induction of γH2AX foci in cultured human cells reveals that SiR-Hoechst induces DNA damage responses and G2 arrest at concentrations well below 1 µM. SiR-Hoechst is useful for live cell imaging, but it should be used with caution and at the lowest practicable concentration.

## Introduction

The ability to observe chromatin in living cells is invaluable in cell biology, allowing individual cells to be followed within cultures or tissues, and the fate of chromosomes within cells to be tracked (for example during cell division or apoptosis). Cell permeable fluorescent DNA dyes that allow chromatin to be visualized in many cell types without the need for introducing exogenous fluorescent proteins by transfection are therefore appealing. However, DNA dyes such as Hoechst 33342 are known to cause DNA damage, particularly during DNA replication, and so alter the behaviour of the cells under observation. Such damage may be brought about by disruption of cellular processes because of binding of the dye to DNA, by photochemical toxicity caused by excitation of the fluorescent molecule, or by a combination of the two^1–3^. A recently developed cell-permeable DNA probe, SiR-Hoechst (also known as SiR-DNA)^4^, is reported not to cause toxicity and has been commercialized, widely publicized, and adopted by numerous laboratories for live cell imaging^5–37^. SiR-Hoechst has some clear advantages: it is selective for DNA; its fluorescence is enhanced upon DNA binding; it is excited by far-red light, avoiding damage caused by the UV light required for traditional Hoechst dyes; and it is compatible with live-cell super-resolution microscopy. However, although in the original report there was little detectable effect on mitotic progression (over 3.4 h) or proliferation of transformed HeLa cells (over 24 h), no detailed analyses of cell cycle progression or specific measurements of DNA damage were carried out in either transformed or in non-transformed cell lines^4^.

## Results and Discussion

During a normal cell cycle, Cyclin B1 accumulates in the cytoplasm and at centrosomes during G2, enters the nucleus several minutes before nuclear envelope breakdown at the onset of mitosis, and then is degraded during mitotic exit^38,39^. In transformed cell lines such as U2OS, DNA damage prevents the nuclear import of Cyclin B1 and cells arrest in G2 with high levels of cytoplasmic Cyclin B1^40–42^. By contrast, in non-transformed cell lines such as hTert-immortalized RPE1, Cyclin B1 is imported into the nucleus in a p21-dependent manner during G2 in response to DNA damage, and accumulation of Cyclin B1 at centrosomes remains low^41–45^. Hours later, Cyclin B1 is degraded in the absence of mitosis, and the cells become senescent^41,42,45^.

**Figure 1:**
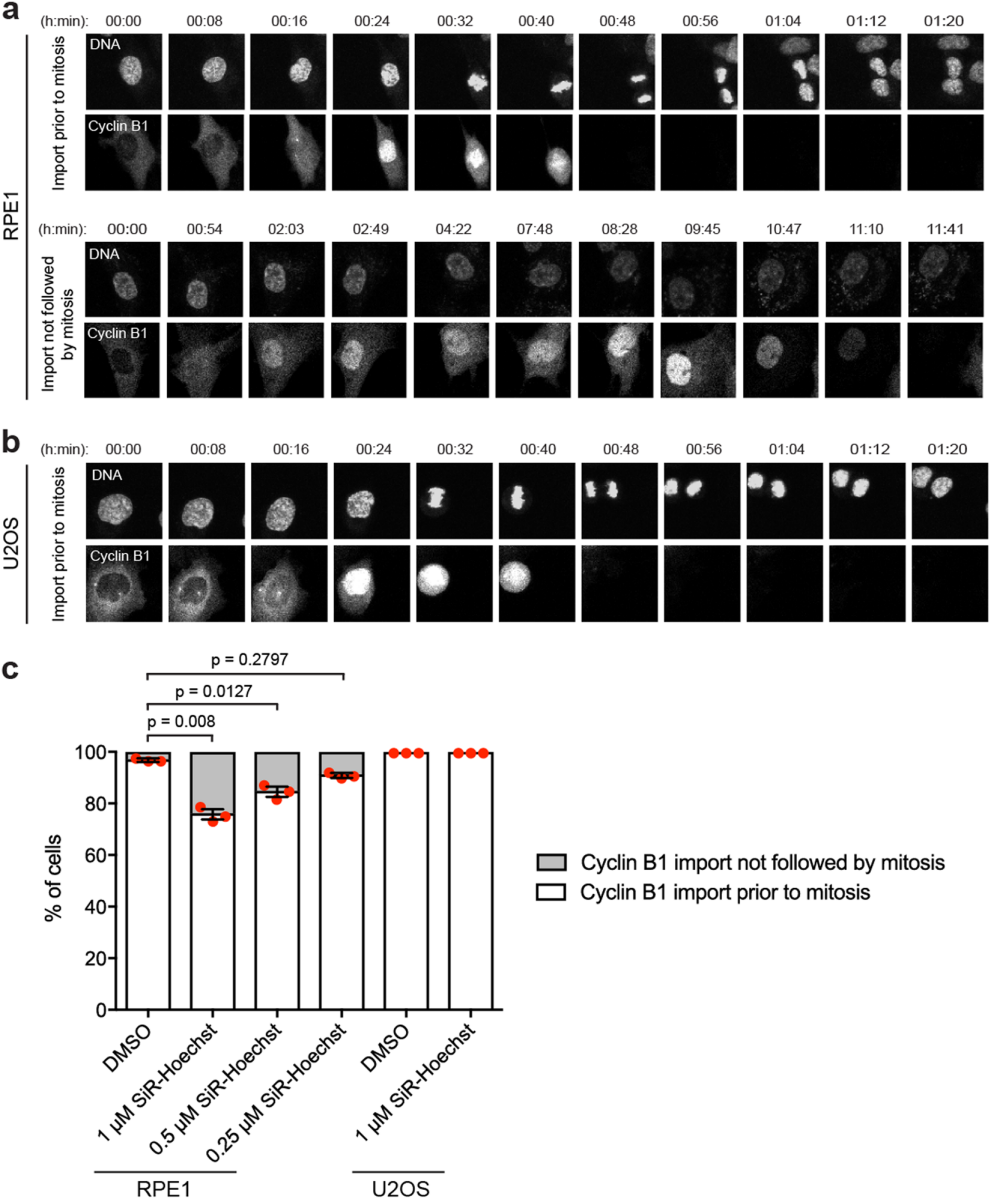
Live imaging in the presence of SiR-Hoechst causes nuclear retention of Cyclin B1 in RPE1 cells, independent of mitosis. (**a**) Asynchronous RPE1 cells expressing Cyclin B1-EYFP were treated with DMSO or different concentrations (1 µM, 0.5 µM, 0.25 µM) of SiR-Hoechst for 2 h prior to live imaging for 18–19 h in the continued presence of DMSO or SiR-Hoechst. Representative movie stills illustrate the two cell fates observed. Also see Supplemental Movies 1 to 4. (**b**) Asynchronous U2OS cells expressing Cyclin B1-EYFP were treated with DMSO or 1 µM SiR-Hoechst for 2 h prior to live imaging for 18 h in the continued presence of SiR-Hoechst. In addition, all U2OS cells were treated with 10 µM Verapamil to inhibit efflux pumps. Representative movie stills illustrate the cell fate observed. Also see Supplemental Movies 5 and 6. (**c**) For cells showing nuclear import of Cyclin B1, the fates indicated in (a,b) were quantified in three independent experiments. Between 152 and 623 cells were counted per treatment. Error bars indicate mean ± SEM (n = 3). Statistical significance was determined using unpaired t-tests.

To track Cyclin B1 localisation in response to SiR-Hoechst, we used RPE1 and U2OS cell lines that express Cyclin B1-EYFP from its endogenous locus^46,47^. We treated RPE1 and U2OS cells with a range of SiR-Hoechst concentrations^4^, and observed the localisation of both Cyclin B1-EYFP and SiR-Hoechst by live imaging for 18 to 19 h. In RPE1 cells we observed two major cell fates: (i) timely Cyclin B1 import prior to mitosis, and (ii) Cyclin B1 import followed by later degradation in the absence of mitosis, reflecting arrest in G2 (Fig. 1a). Among control cells treated with DMSO that imported Cyclin B1 into the nucleus, 3% displayed non-mitotic import of Cyclin B1 (see example Supplemental Movie 1), but this was significantly increased to 24% in cells treated with 1 µM SiR- Hoechst (Supplemental Movie 2, Fig. 1c). An increase in the percentage of RPE1 cells showing non-mitotic import of Cyclin B1 was also seen at 0.5 µM and 0.25 µM SiR-Hoechst, though the magnitude of this effect declined as the concentration was decreased (Fig. 1c; Supplemental Movies 3 and 4). As expected, the transformed cell line U2OS did not display non-mitotic nuclear import of Cyclin B1, in either controls or after treatment with 1 µM SiR-Hoechst, but Cyclin B1 accumulated in the cytoplasm over longer periods in the presence of SiR-Hoechst (Fig. 1b,c; Supplemental Movies 5 and 6). Therefore, both RPE1 and U2OS cells show evidence of an arrest or delay in G2 in response to SiR-Hoechst.

**Figure 2:**
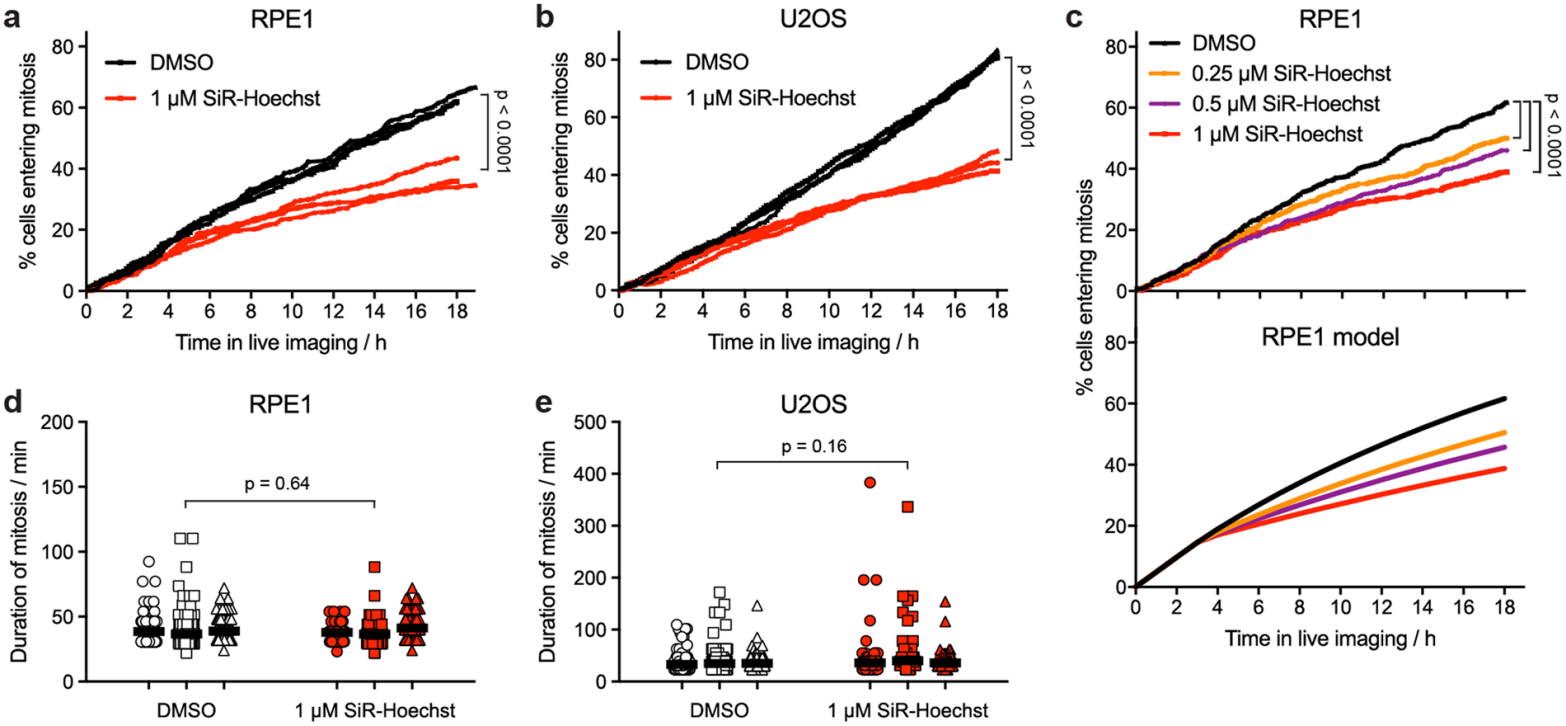
Live imaging in the presence of SiR-Hoechst delays cell division but does not alter the duration of mitosis. (**a**) Asynchronous RPE1 cells expressing Cyclin B1-EYFP were treated with DMSO or 1 µM SiR-Hoechst for 2 h then imaged for 18–19 h in the continued presence of DMSO or SiR-Hoechst. Mitotic entry was defined as the first live imaging frame in which mitotic nuclear import of Cyclin B1 was detected. Between 265 and 453 total cells were counted per treatment. The results of three independent experiments are shown. Statistical significance was determined from the combined data using Kaplan-Meier curve analysis and a log rank test. (**b**) As for (a), but for U2OS cells expressing Cyclin B1-EYFP. Between 561 and 695 total cells were counted per treatment (n = 3). (**c**) Top panel. As for (a), but RPE1 cells expressing Cyclin B1-EYFP were treated with DMSO, 0.25, 0.5 or 1 µM SiR-Hoechst. The combined results of 4 fields of view from two independent experiments are shown. Between 689 and 889 total cells were counted per treatment. Bottom panel. Theoretical time courses of RPE1 cell mitotic entry derived from the mathematical model using the following parameters: normal length of cell cycle, 20.6 h (derived from Supplemental Fig. 2); length of G2 plus M, 5.2 h^49^; imaging damage coefficient, 0.6; pre-incubation time in SiR-Hoechst, 2 h; percentage of cells arresting in G2 (determined from imaging of non-mitotic G2 import) in DMSO, 2.1%; in 0.25 µM SiR-Hoechst, 6.9%; in 0.5 µM SiR-Hoechst, 7.4%; in 1 µM SiR-Hoechst, 9.9%. (**d**) The duration of mitosis for the RPE1 cells quantified in (a) was defined as described in Methods. Between 114 to 266 mitotic cells were quantified per treatment, in three independent experiments. Horizontal bars indicate the mean duration of mitosis. Statistical significance was determined from the combined data using a paired t-test (n = 3). (**e**) As for (d), but for U2OS cells expressing Cyclin B1-EYFP. Between 227 and 544 mitotic cells were quantified per treatment (n = 3).

As an alternative way to monitor cell cycle delay in both RPE1 and U2OS cells, we counted the number of cells entering mitosis during live imaging. The presence of 1 µM SiR-Hoechst caused the number of RPE1 and U2OS cells entering mitosis to drop by more than 40% over the period of imaging (Fig. 2a,b). This outcome was concentration-dependent, but declines in mitotic entry were also observed at 0.5 and 0.25 µM SiR-Hoechst (Fig. 2c). To estimate the extent of the delay, we developed a simple mathematical model of cell cycle progression in which SiR-Hoechst caused DNA damage in S phase, leading to either cell cycle arrest or delay in S/G2. For RPE1, cells arresting in G2 were defined as those that imported Cyclin B1 in the absence of mitosis during live imaging (approximately 10% of all cells at 1 µM SiR-Hoechst). The rates of mitotic entry observed at 0.25, 0.5 and 1 µM SiR-Hoechst were best modelled when the remaining non-arresting cells were delayed in S/G2 for an average of 8, 14 or 26 hours, respectively (Fig. 2c; Supplemental Fig. 1a). For U2OS cells, which do not import Cyclin B1 into the nucleus in response to DNA damage, the model suggested that S/G2 was extended by an average of 33 h at 1 µM SiR-Hoechst (Supplemental Fig. 1b). In both the results and the models, the effect of SiR-Hoechst was most pronounced after 4 h or more of live imaging (Fig. 2a–c; Supplemental Fig. 1), reflecting the ability of cells that are already in G2 when SiR-Hoechst is added to proceed relatively unimpeded into mitosis. Reducing the intensity of laser illumination during imaging did not prevent the detrimental effect of SiR-Hoechst on RPE1 cells (Supplemental Fig. 2). Although cells in mitosis itself have sometimes been considered particularly sensitive to damage during live imaging^4,48^, for those cells that divided, SiR-Hoechst had little influence on the duration of mitosis in either RPE1 or U2OS cells (Fig. 2d,e). These findings show that, during live imaging, SiR-Hoechst can prevent a significant portion of cells from entering mitosis, and suggest that it induces a DNA damage response that delays or arrests cell cycle progression in G2. In addition, they demonstrate that normal mitotic duration cannot be taken as evidence that cells are unaffected by the dye.

**Figure 3:**
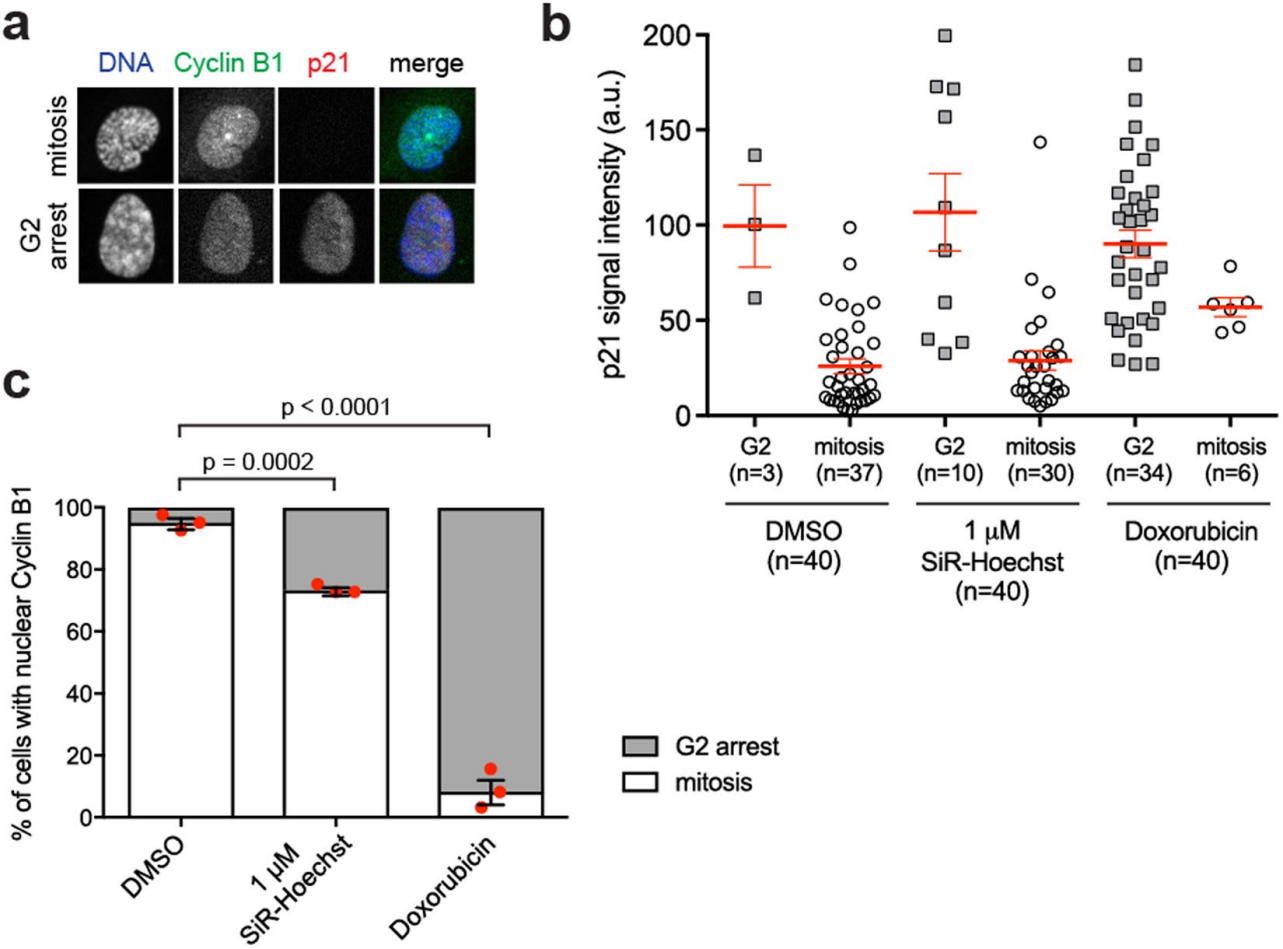
SiR-Hoechst causes non-mitotic nuclear Cyclin B1 import in the absence of imaging. (**a**) Asynchronous RPE1 cells expressing Cyclin B1-EYFP were treated with DMSO, 1 µM SiR-Hoechst or 0.5 µM Doxorubicin for 20 h before fixation. Fixed cells were co-stained with Hoechst 33342 (DNA), anti-GFP (Cyclin B1-EYFP) and anti-p21 antibodies. Representative images show G2 or mitotic cells that imported Cyclin B1 into nucleus. (**b**) Asynchronous RPE1 cells expressing Cyclin B1-EYFP were treated and stained as in (a). Cells with nuclear Cyclin B1 were classified as in G2 (cells with uncondensed nuclear chromatin and without centrosomal Cyclin B1) or in mitosis (cells with clearly condensing chromosomes and/or centrosomal Cyclin B1), and the intensity of nuclear p21 staining was quantified in 40 cells per treatment in one experiment. Means ± SEM are shown. Note that cells classified as arrested in G2 typically have higher levels of p21 than cells entering mitosis. (**c**) The cell fates indicated in (a,b) were quantified in 40 cells per treatment in three independent experiments. Error bars indicate mean ± SEM (n = 3). Statistical significance was determined using unpaired t-tests.

To determine if this effect of SiR-Hoechst on living RPE1 cells requires light exposure during microscopy, we conducted experiments in which cells were treated with SiR-Hoechst for 20 h in the absence of imaging, followed by fixation and immunofluorescence microscopy. Among cells that had nuclear Cyclin B1, we then determined the proportion that was in G2 or mitosis. Cells with uncondensed chromatin and nuclear but not centrosomal Cyclin B1, and typically with nuclear p21, were classified as in G2. Cells with nuclear Cyclin B1 and clearly condensing chromosomes and/or centrosomal Cyclin B1, typically without nuclear p21, were classified as in mitosis (see Fig. 3a,b). Among control DMSO-treated cells with nuclear Cyclin B1, 5% were in G2, while the majority (95%) were in prophase of mitosis (Fig. 3c). When treated with the known DNA damaging agent Doxorubicin, the majority (92%) of cells with nuclear Cyclin B1 were in G2. Notably, 1 µM SiR-DNA treatment also caused a significant increase, to 27%, in the percentage of cells with nuclear Cyclin B1 that were in G2 (Fig. 3c). These data show that binding of SiR-Hoechst to DNA is sufficient to cause nuclear Cyclin B1 retention in G2 in the absence of imaging, although laser exposure may exacerbate the effect (see Supplemental Fig. 2).

**Figure 4:**
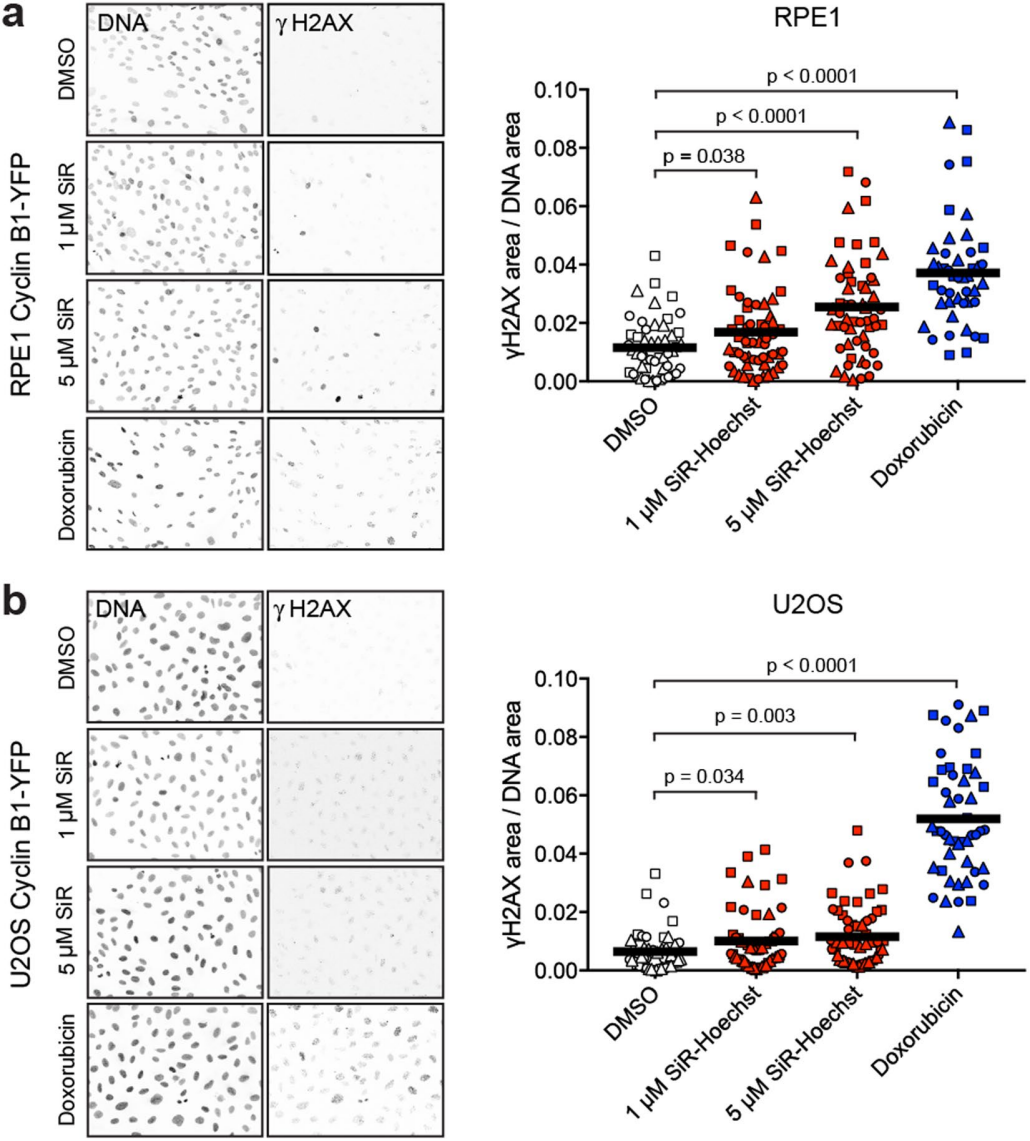
SiR-Hoechst can cause DNA damage in the absence of imaging. (**a**) Asynchronous RPE1 cells expressing Cyclin B1-EYFP were treated with DMSO, 1 or 5 µM SiR-Hoechst, or 0.5 µM Doxorubicin for 20 h, followed by fixation. Fixed cells were co-stained with Hoechst 33342 (DNA) and anti-γH2AX antibodies. The proportion of pixels that exceeded a fixed threshold of γH2AX intensity was then determined in three independent experiments (circle, square, triangle symbols) and reported as the (area containing γH2AX staining)/(area containing DNA staining, i.e. nuclei). Approximately 18 fields were measured per condition in each experiment. Means are indicated by horizontal bars. Statistical significance was determined using unpaired t-tests (n = 44 to 56). (**b**) Asynchronous U2OS cells expressing Cyclin B1-EYFP were treated and analysed as in (a). Statistical significance was determined using unpaired t-tests (n = 49 to 58). In addition, U2OS cells were treated with 10 µM Verapamil throughout to inhibit efflux pumps.

Finally, we measured the induction of the DNA damage marker γH2AX (H2AX S139ph) to determine if SiR-Hoechst caused DNA damage in cells. As expected, Doxorubicin strongly induced γH2AX in both RPE1 and U2OS cell lines (Fig. 4a,b). At 1 µM, SiR-Hoechst caused a small increase in γH2AX foci in both RPE1 and U2OS cells and, at 5 µM, more clearly induced γH2AX foci (Fig. 4a,b) particularly in RPE1 cells. Therefore, SiR-Hoechst may cause DNA damage even in the absence of imaging.

By the measures of non-mitotic nuclear import of Cyclin B1, inhibition of mitotic entry and the induction of γH2AX foci, it is clear that SiR-Hoechst causes changes in cell cycle progression, probably at least in part due to DNA damage. U2OS cells, as well as the widely-used non-transformed cell line RPE1, are susceptible to a substantial G2 delay or arrest, even well below the manufacturer’s recommended concentration of 1 µM. A majority of published studies use SiR-Hoechst at a concentration of 0.5 µM or higher (Supplemental Table 1), but we find that it is not a benign DNA stain in living cells. Our results show that concentrations below 0.25 µM should be used to minimize cell cycle progression defects. These concentrations are sufficient for high-quality imaging, at least by laser scanning confocal microscopy. Importantly, because the duration of mitosis can be unaffected under conditions in which the rate of mitotic entry is substantially reduced, apparently normal chromosome congression and segregation cannot be taken as evidence that SiR-Hoechst is not altering the biology of the cells under observation. We hope that the observations and simple mathematical model reported here will help others to optimize suitable conditions for their own experiments. SiR-Hoechst is an excellent addition to the cell biologist’s toolbox, but we urge researchers to use it at the lowest possible concentration.

## Methods

### Cell culture

U2OS or hTert-RPE1 cells that express Cyclin B1-EYFP from its endogenous locus^46,47^ were cultured in DMEM or DMEM/F12 media (Sigma), respectively, supplemented with 10% Foetal Bovine Serum (FBS) (Labtech) and penicillin/streptomycin (Sigma) at 37 °C and 5% CO_2_.

### Time-lapse microscopy

Cells were seeded in 4-chamber glass-bottom dishes (Greiner Bio One) 24 h before imaging. Two hours before imaging, cells were loaded with SiR-Hoechst (SiR-DNA; Spirochrome) or vehicle control (DMSO). In addition, U2OS cells were treated with 10 µM Verapamil (Spirochrome) to inhibit dye efflux pathways and enable homogenous DNA staining^4^. Time-lapse imaging was then carried out at 37 °C, 5% CO_2_ for 18 to 18.9 hours using an inverted Nikon A1R confocal microscope. Observations were performed throughout using a Plan Apo 20x 0.75 NA air objective with an excitation pixel dwell time of 2.4 µs. Images of 1024 × 1024 pixels were captured with a pixel size of 0.63 µm. Confocal pinholes were set to capture approximately 2 µm thick optical sections for each channel. Sections were taken every 5 µm over 20 µm (5 sections in total). Volumes were captured approximately every 8 minutes. Channel observation conditions were as follows. For EYFP: excitation wavelength 514 nm; laser power recorded at object of 47 µW; GaSaP detector gain of 43/255. For SiR-Hoechst: excitation wavelength 640 nm; laser power recorded at object of 190 µW; standard PMT detector gain of 81/255. Where stated, alternative settings for SiR-Hoechst were used: excitation wavelength 640 nm; laser power recorded at object of 86 µW; standard PMT detector gain of 81/255. Power measurements were made in the same imaging conditions using a Thorlabs S170c microscope slide photodiode sensor and PM100D meter. All images were manually analysed and quantified using NIS-Elements (Nikon) software. The time of mitotic entry was determined as the first movie frame in which Cyclin B1-EYFP rapidly entered the nucleus prior to cell division visualised by transmitted light imaging. The duration of mitosis was defined as the time elapsed between this frame and the first frame in which Cyclin B1-EYFP was eliminated in anaphase. Statistical analyses were carried out using Prism 7 (GraphPad Software).

### Cell Cycle Model

To model the effect of SiR-Hoechst on the cell cycle, we assumed that dye-induced DNA damage is caused during DNA replication (S phase) and that this results in cell cycle arrest or delay in S or G2 phases. In the model, cells that are in G2 or mitosis at the time of SiR-Hoechst addition can proceed unimpeded into and through mitosis (first term in the equation below). Cells that pass through S phase during imaging can have one of three fates. Two populations suffer delays in S/G2 of *t*_*x*_ or *t*_*y*_ hours (where *t*_*x*_ or *t*_*y*_ ≥ 0, and *t*_*x*_ or *t*_*y*_ = *∞* when arrested), represented by the second and third terms in the equation. Another population is unaffected by SiR-Hoechst (final term in equation). Other parameters are as follows: *t*_*c*_ is the normal duration of one cell cycle (determined using imaging conditions in which % mitotic entry increased linearly for the entire duration of imaging); *t*_*G2M*_ is the normal length of time spent in G2 and M phases (obtained from the literature)^46,49^; *t*_*p*_ is the time in hours during which SiR-Hoechst was pre-incubated with cells prior to imaging; *t*_*i*_ is the number of hours since imaging began; *c* is a coefficient accounting for progressive damage caused by imaging in the absence of SiR-Hoechst; *x* and *y* are the fractions of cells suffering a delay of t_x_ and t_y_ hours, respectively (where *x* + *y* ≤ 1).

$$\begin{array}{rcl} \% \,cells\,entering\,mitosis & = & \frac{100(min(max({t}_{G2M}-{t}_{p},\,0),\,{t}_{i}))}{{t}_{c}}\\  &  & +\,\frac{100x({t}_{i}-max({t}_{G2M}-{t}_{p},\,0),\,0)}{{t}_{c}+{t}_{x}+c{t}_{i}}\\  &  & +\,\frac{100y({t}_{i}-max({t}_{G2M}-{t}_{p},\,0),\,0)}{{t}_{c}+{t}_{y}+c{t}_{i}}\\  &  & +\,\frac{100(1-x-y)({t}_{i}-max({t}_{G2M}-{t}_{p},\,0),\,0)}{{t}_{c}+c{t}_{i}}\end{array}$$

### Immunofluorescence microscopy

Cells were seeded on glass coverslips coated with poly-lysine and, after 24 h, treated with SiR-Hoechst (SiR-DNA; Spirochrome), doxorubicin hydrochloride (Sigma) or DMSO for 20 h before fixation with 2% (w/v) paraformaldehyde (Thermo Fisher Scientific) in PBS for 10 minutes at room temperature. After washing three times with PBS, cells were incubated in 0.5% (v/v) Triton X-100 in PBS for 10 minutes, washed three times with PBS and blocked in BlockAid solution (Thermo Fisher Scientific) for 1 h at room temperature. Cells were stained for 18 h at 4 °C with the following primary antibodies diluted in BlockAid solution: mouse anti-GFP monoclonal antibody (Abcam, ab1218), mouse anti-phospho-Histone H2AX Ser-139 (γH2AX) monoclonal antibody (Merck Millipore, 05–636), rabbit anti-p21 polyclonal antibody (Abcam, ab109520). After washing three times with 0.05% (v/v) Triton X-100 in PBS, cells were stained with the following secondary antibodies diluted in BlockAid solution for 1 h at 37 °C: goat anti-rabbit Alexa Fluor 488 or Alexa Fluor 594, donkey anti-mouse Alexa Fluor 488 or Alexa Fluor 594 (Jackson ImmunoResearch). After washing, cells were stained with 2 µg/ml Hoechst 33342 (Thermo Fisher Scientific, H2570) in PBS for 5 min at room temperature, before washing with PBS and mounting using Prolong Diamond Antifade Mountant (Thermo Fisher Scientific, P36961). Images were obtained using a Zeiss Axio Imager, equipped with Apotome, 20x 0.8 NA air objective, LED light source, AxioCam MR R3 camera and ZEN (Zeiss) software. To obtain γH2AX/DNA signals, images of approximately 18 fields per treatment in each experiment on a coverslip were captured. DNA and γH2AX signals were quantified using Photoshop CS6 by determining the number of pixels within each image exceeding a fixed threshold intensity for γH2AX signal (“fuzziness” level 80) and DNA signal (level 40). The signal was then expressed as a ratio of the area containing γH2AX staining over the area containing DNA signal. The signal intensity of nuclear p21 was quantified in G2 and prophase cells using ImageJ. Statistical analyses were carried out using Minitab 17 (Minitab Inc.) and Prism 7 (GraphPad Software).

## Electronic supplementary material

Supplemental Information

Supplemental Movie 1

Supplemental Movie 2

Supplemental Movie 3

Supplemental Movie 4

Supplemental Movie 5

Supplemental Movie 6
