# Supplementary material for: The live cell DNA stain SiR-Hoechst induces DNA damage responses and impairs cell cycle progression
Source: Sci Rep. 2018 May 21;8:7898. doi: 10.1038/s41598-018-26307-6 (PMC5962532; doi:10.1038/s41598-018-26307-6)
Supplement: Supplementary file 1 — Supplemental Information [file 41598_2018_26307_MOESM1_ESM.pdf]

Supplemental Information

The live cell DNA stain SiR-Hoechst induces DNA damage responses and impairs cell cycle progression

Onur Sen[1], Adrian T. Saurin[2] and Jonathan M.G. Higgins[1*]

[1] Cell Division Biology Group, Institute for Cell and Molecular Biosciences, Newcastle University, Medical School, Framlington Place, Newcastle upon Tyne, NE2 4HH, UK.
[2] Division of Cancer Research, School of Medicine, Jacqui Wood Cancer Centre, Ninewells Hospital and Medical School, University of Dundee, Dundee, DD1 9SY, UK.

* Correspondence and requests for materials should be addressed to J.M.G.H. (email: jonathan.higgins@ncl.ac.uk)

Supplemental Table S1

Concentrations of SiR-Hoechst used in published live imaging studies

SiR-Hoechst concentration (µM)	References
0.05	21,32
0.1	18,23,29,34
0.1-0.2	8,36
0.2	24
0.3	30
0.5	9,11,16,28,33
1	7,15,20,35,37
2	10,27
50	22
90*	13
1000	25
Not stated	5,6,12,14,17,19,26,31

* 1 nl of a 90 µM SiR-Hoechst solution was microinjected into live zebrafish embryos, and is likely to have become diluted approximately 100-fold within the cell(s).

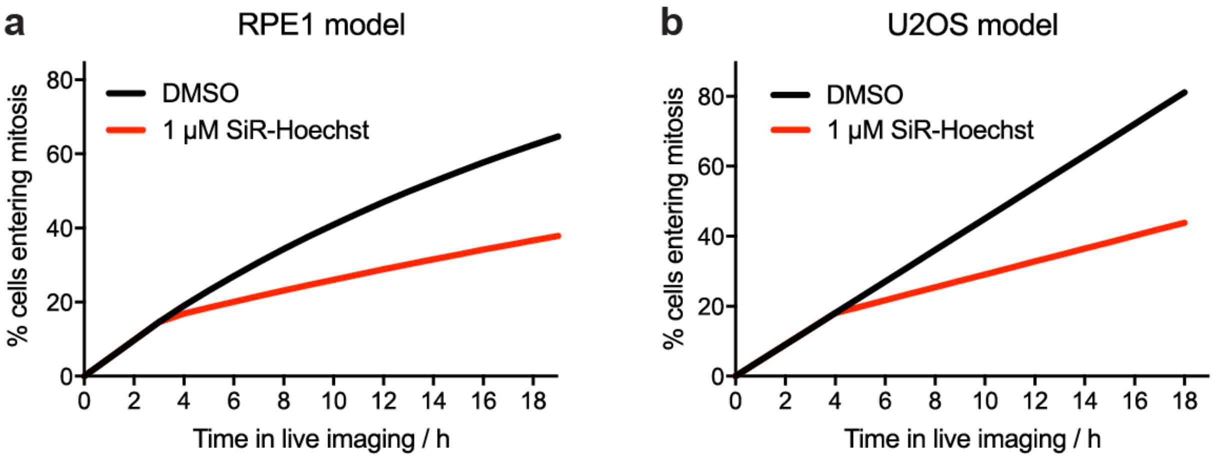

Supplemental Figure S1. Time course of mitotic entry using a mathematical model of cell cycle progression in the presence of DNA damage.

The model, described in more detail in Methods, assumes that SiR-Hoechst induces DNA damage during replication in S phase, followed by cell cycle delay or arrest in S/G2.

a. To model the progression of RPE1 cells in Figure 1a, we used the following parameters: normal length of cell cycle, 20.6 h (derived from Supplemental Figure 2); length of G2 plus M, 5.2 h (ref 49); imaging damage coefficient, 0.58; pre-incubation time in SiR-Hoechst, 2 h; percentage of cells arresting in G2 in DMSO, 2.1%, and in SiR-Hoechst, 9.1% (determined from non-mitotic G2 import). The model suggests that, for cells that do not arrest in G2, 1 μM SiR-Hoechst causes a mean S/G2 delay of 33 h.

b. To model the progression of U2OS cells in Figure 1b, we used the following parameters: normal length of cell cycle, 22.2 h (derived from Figure 2b); length of G2 plus M, 6 h (ref 46); imaging damage coefficient, 0; pre-incubation time in SiR-Hoechst, 2 h; percentage of cells arresting in G2, 0% (non-mitotic G2 import is not observed in U2OS cells). The model suggests that 1 μM SiR-Hoechst causes a mean S/G2 delay of 32 h.

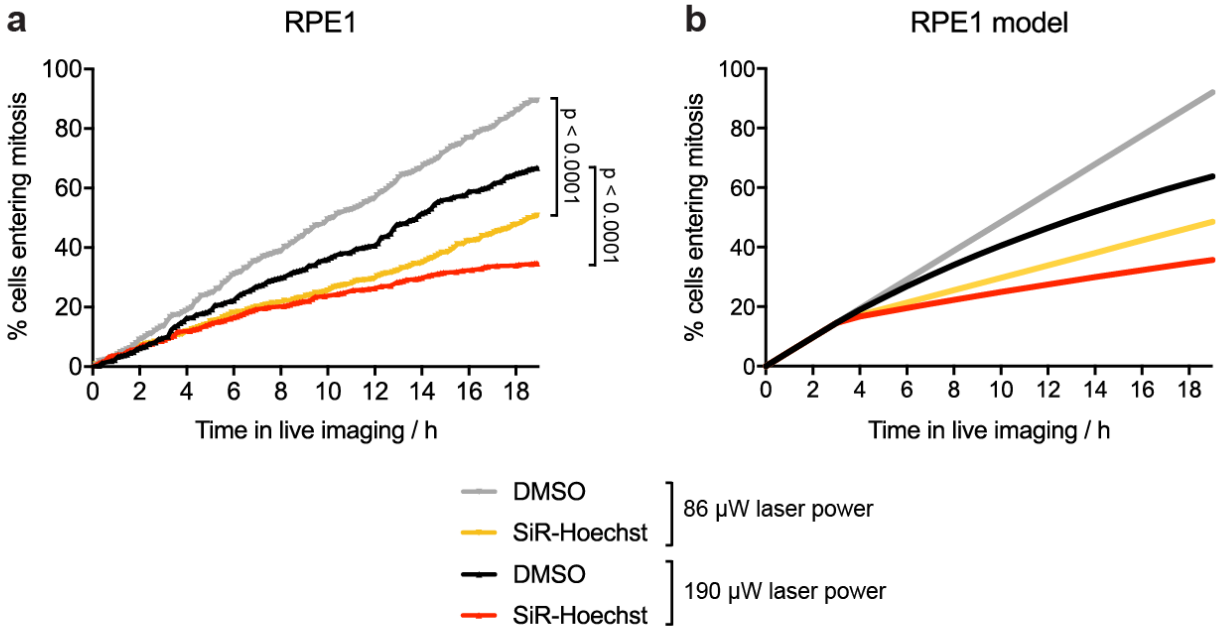

Supplemental Figure S2. Live imaging in the presence of SiR-Hoechst prevents cell division at different laser illumination intensities.

a. Asynchronous RPE1 cells expressing Cyclin B1-EYFP were treated with DMSO or 1 μM SiR-Hoechst for 2 h then imaged for 18.9 h at two different 640 nm laser intensities (*i.e.* SiR-Hoechst excitation) in the continued presence of DMSO or SiR-Hoechst. The time at which cells entered mitosis was defined as described in Methods. In both cases, approximately half as many cells entered mitosis in the presence of 1 μM SiR-Hoechst as in its absence. Laser powers recorded at the object are given (see Methods). Between 370 and 501 cells were counted per treatment. Statistical significance was determined using Kaplan-Meier curve analysis and log rank test.

b. Time course of mitotic entry using the mathematical model with the following parameters: normal length of cell cycle, 20.6 h (derived from imaging in DMSO at 86 μW laser power); length of G2 plus M, 5.2 h (ref 49); imaging damage coefficient, 0 at 86 μW and 0.6 at 190 μW laser power; pre-incubation time in SiR-Hoechst, 2 h; percentage of cells arresting at G2 in DMSO, 0.23% at 86 μW and 2.2% at 190 μW; percentage of cells arresting at G2 in 1 μM SiR-Hoechst, 4.8% at 86 μW and 7.9% at 190 μW (determined from imaging of non-mitotic G2 import). The model suggests that, after accounting for the effect of imaging alone, 1 μM SiR-Hoechst produces an additional mean S/G2 delay in non-arrested cells of 25 or 40 h at 86 or 190 μW laser power, respectively. Therefore, imaging appears to exacerbate the effect of SiR-Hoechst.

Supplemental Movies

Movie 1

hTert-RPE1 cells expressing Cyclin B1-EYFP were treated with DMSO as outlined in Figure 1A and EYFP fluorescence was imaged using an inverted Nikon A1R confocal microscope equipped with a 20x 0.75 NA air objective. The interval between frames is approximately 8 min.

Movie 2

hTert-RPE1 cells expressing Cyclin B1-EYFP were treated with 1 μM SiR-Hoechst as outlined in Figure 1A and EYFP and SiR-Hoechst fluorescence was imaged as for Movie 1.

Movie 3

As for Movie 2, but with 0.5 μM SiR-Hoechst.

Movie 4

As for Movie 2, but with 0.25 μM SiR-Hoechst.

Movie 5

As for Movie 1, but with U2OS cells expressing Cyclin B1-EYFP in the presence of DMSO.

Movie 6

As for Movie 5 (U2OS cells), but with 1 μM SiR-Hoechst.
